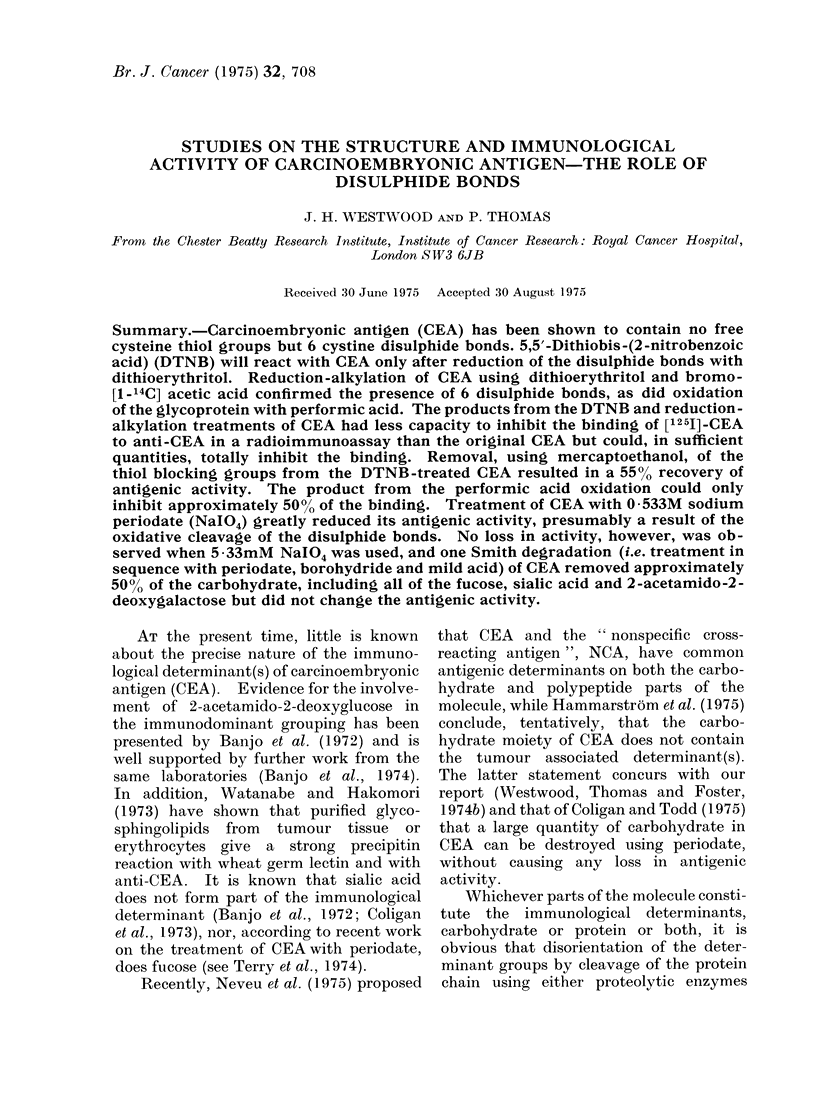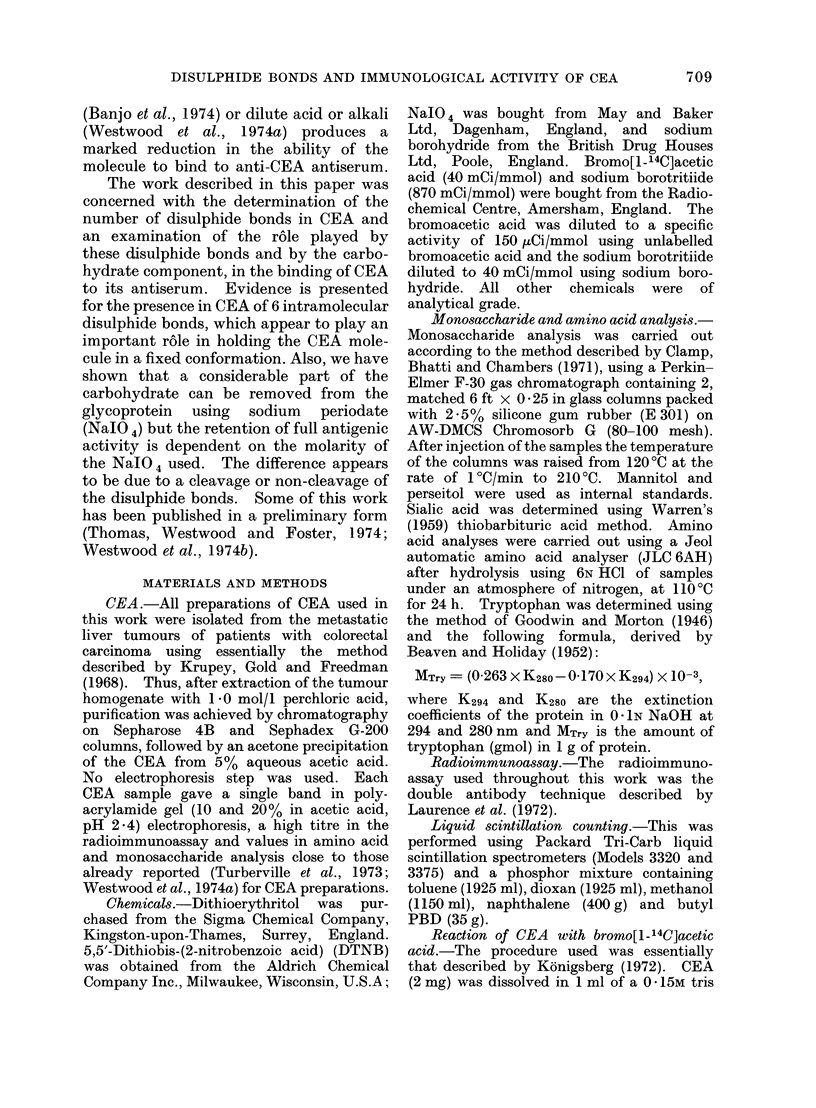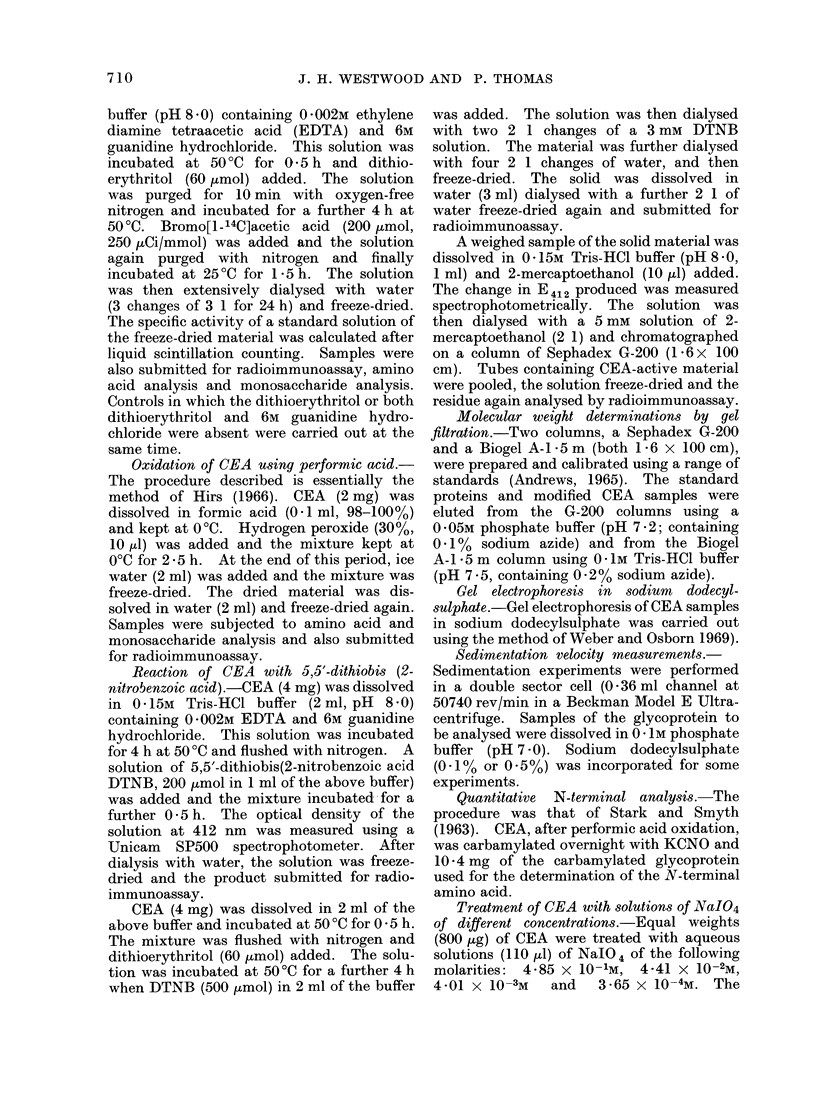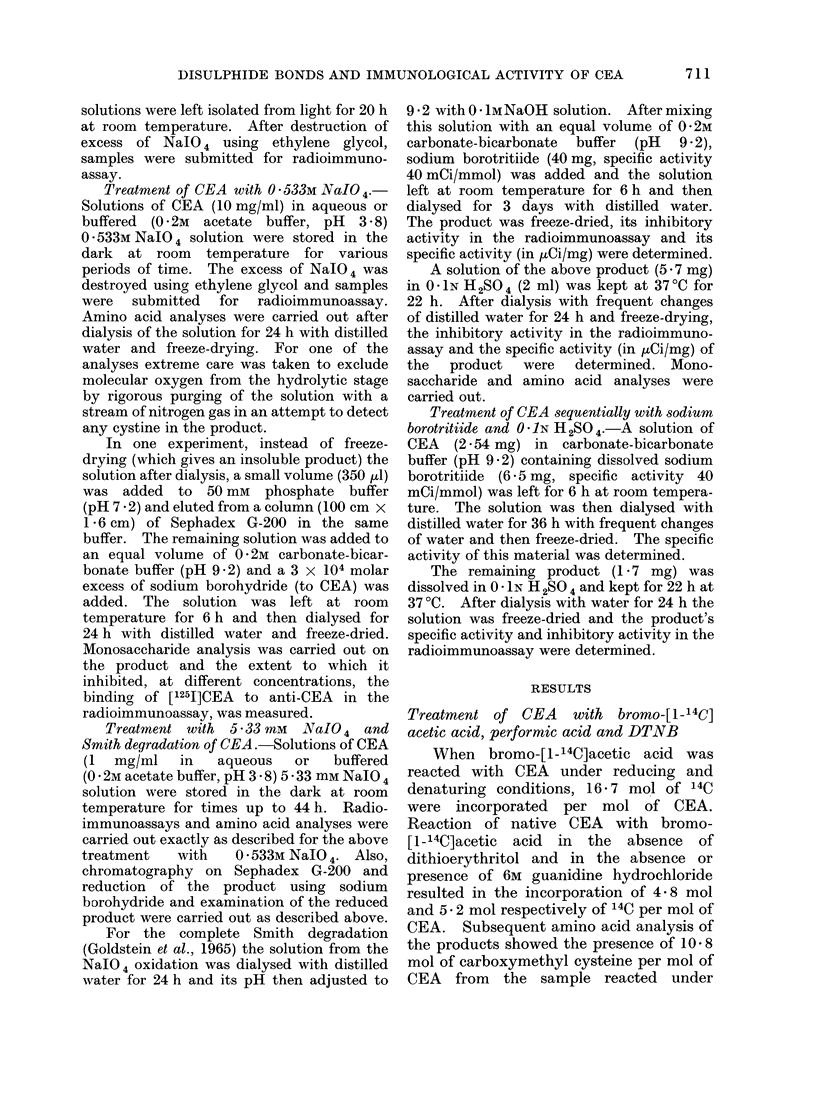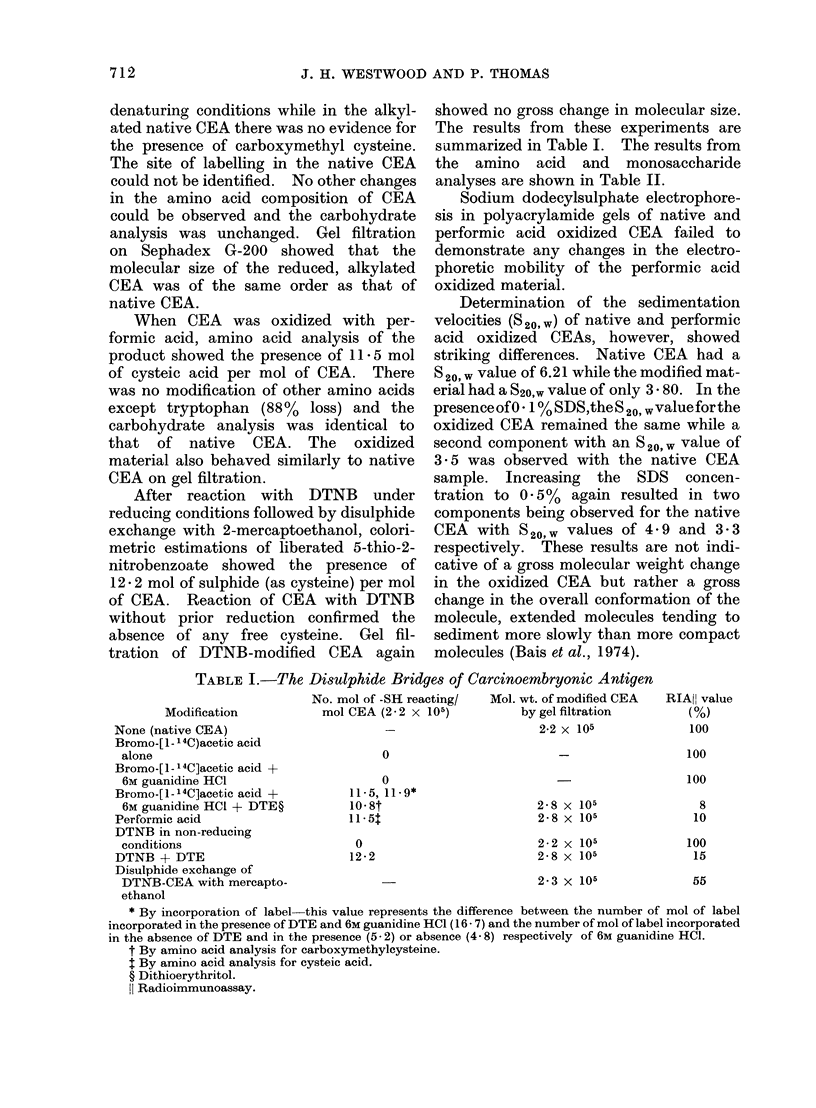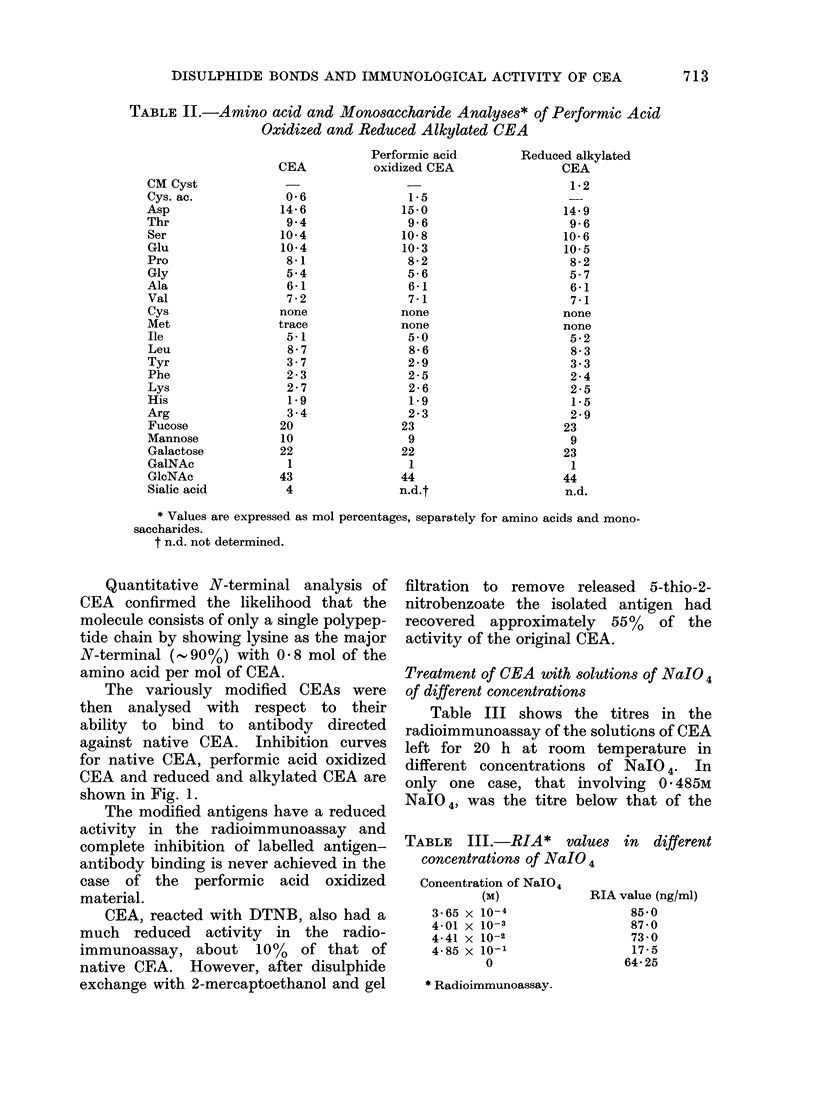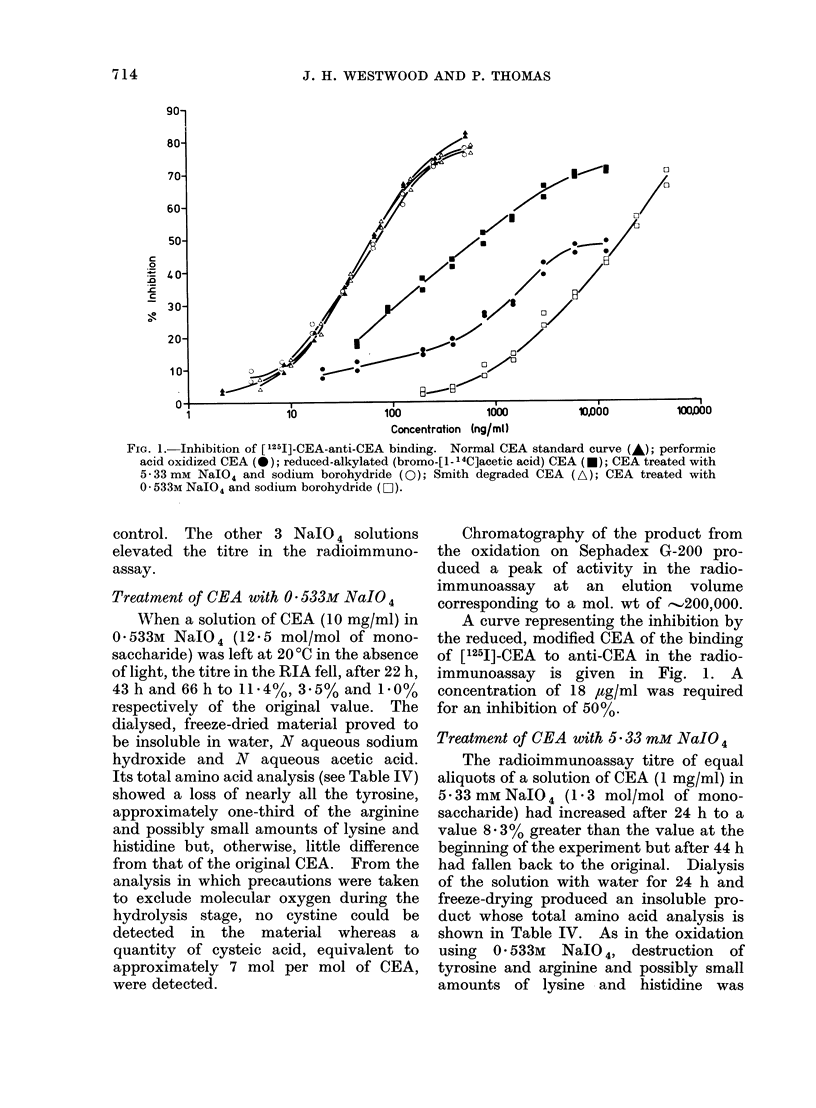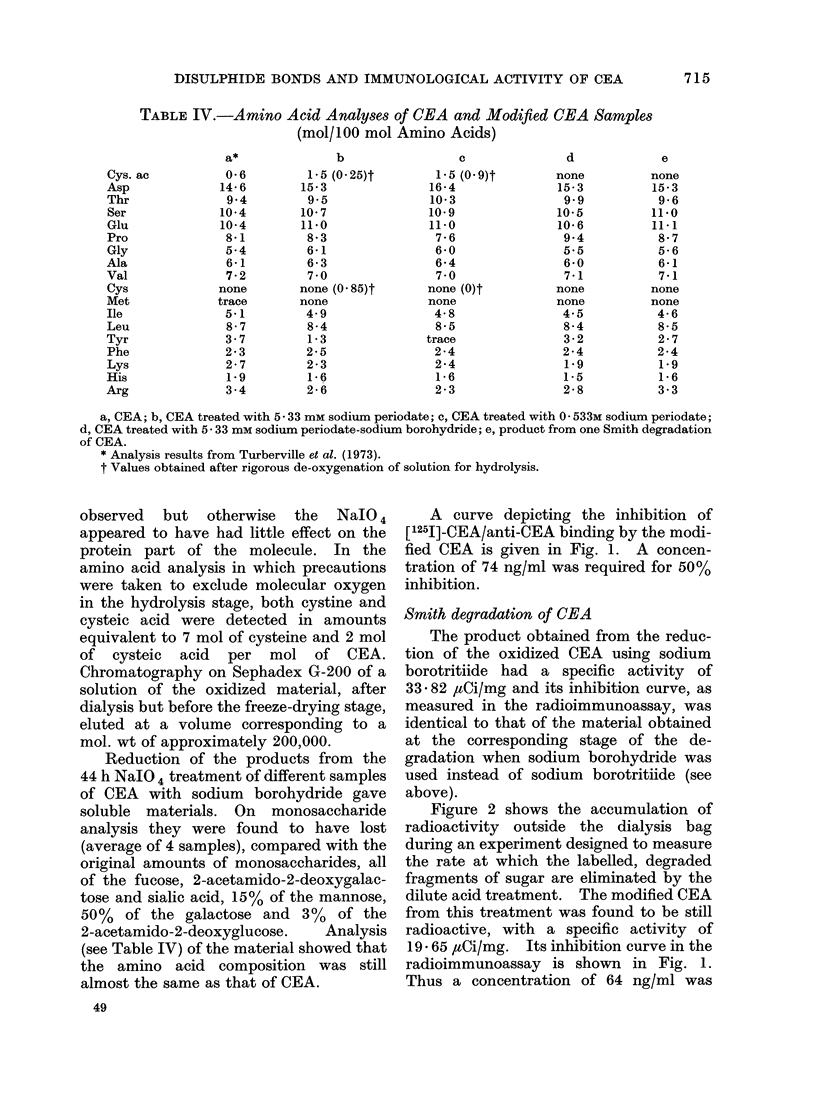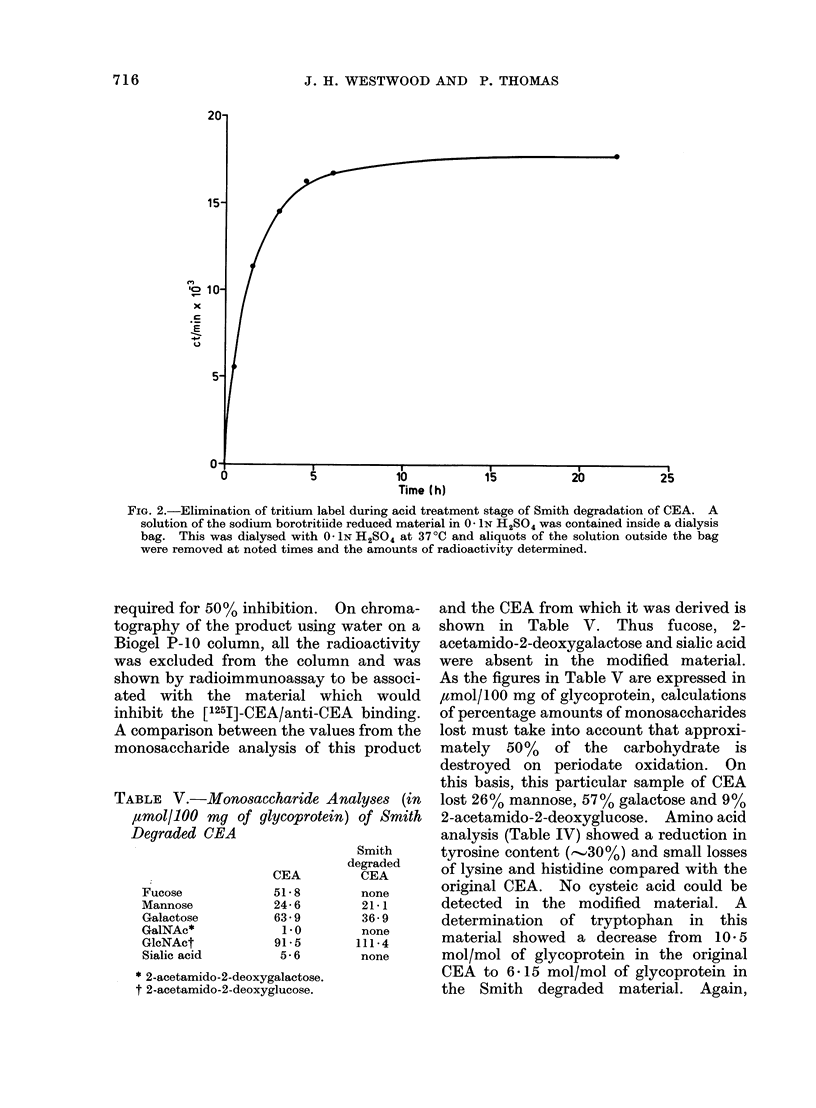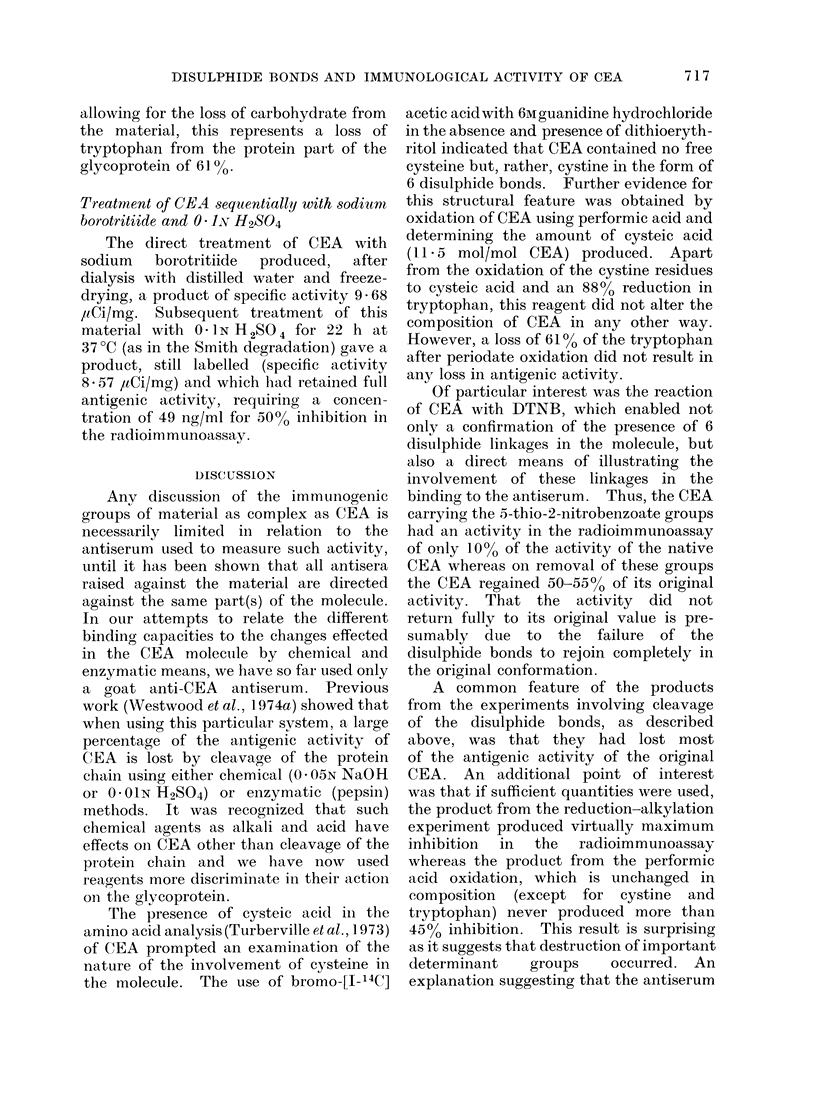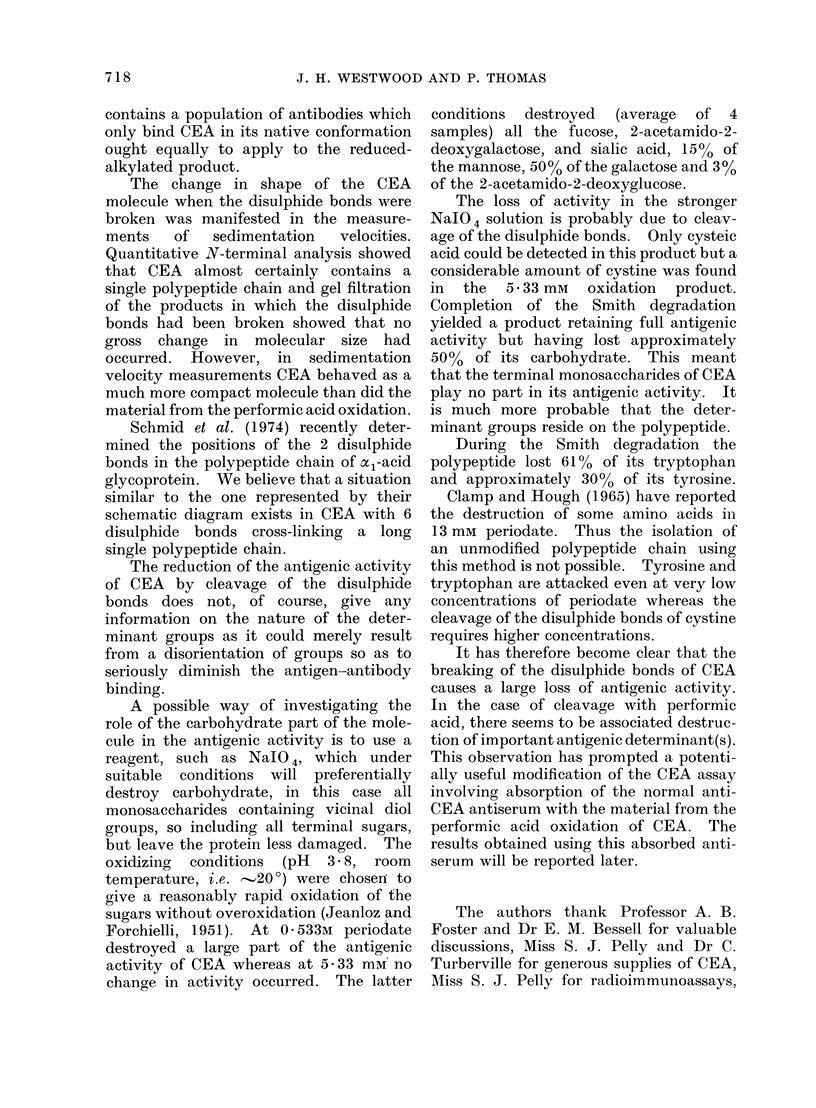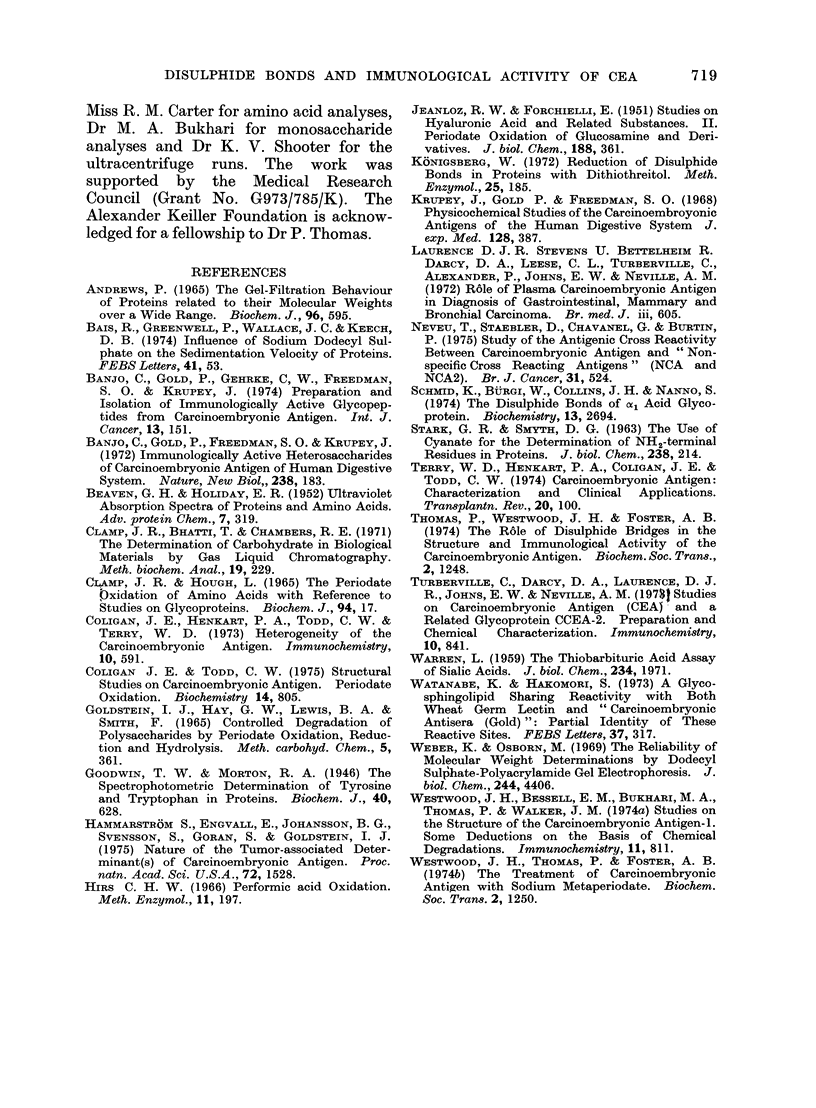# Studies on the structure and immunological activity of carcinoembryonic antigen - the role of disulphide bonds.

**DOI:** 10.1038/bjc.1975.282

**Published:** 1975-12

**Authors:** J. H. Westwood, P. Thomas

## Abstract

Carcinoembryonic antigen (CEA) has been shown to contain no free cysteine thiol groups but 6 cystine disulphide bonds. 5'5-Dithiobis-(2-nitrobenzoic acid) (DTNB) will react with CEA only after reduction of the disulphide bonds with dithioerythritol. Reduction-alkylation of CEA using dithioerythritol and bromo-[1-14C] acetic acid confirmed the presence of 6 disulphide bonds, as did oxidation of the glycoprotein with performic acid. The products from the DTNB and reduction-alkylation treatments of CEA had less capacity to inhibit the binding of [125I]-CEA to anti-CEA in a radioimmunoassay than the original CEA but could, in sufficient quantities, totally inhibit the binding. Removal, using mercaptoethanol, of the thiol blocking groups from the DTNB-treated CEA resulted in a 55% recovery of antigenic activity. The product from the performic acid oxidation could only inhibit approximately 50% of the binding. Treatment of CEA with 0.533M sodium periodate (NaIO4) greatly reduced its antigenic activity, presumably a result of the oxidative cleavage of the disulphide bonds. No loss in activity, however, was observed when 5.33mM NaIO4 was used, and one Smith degradation (i.e. treatment in sequence with periodate, borohydride and mild acid) of CEA removed approximately 50% of the carbohydrate, including all of the fucose, sialic acid and 2-acetamido-2-deoxygalactose but did not change the antigenic activity.


*Br. J. Cancer* (1975) **32**, 708

### STUDIES ON THE STRUCTURE AND IMMUNOLOGICAL ACTIVITY OF CARCINOEMBRYONIC ANTIGEN—THE ROLE OF DISULPHIDE BONDS

J. H. WESTWOOD AND P. THOMAS

*From the Chester Beatty Research Institute, Institute of Cancer Research: Royal Cancer Hospital, London SW3 6JB*

Received 30 June 1975 Accepted 30 August 1975

**Summary.—Carcinoembryonic antigen (CEA) has been shown to contain no free cysteine thiol groups but 6 cystine disulphide bonds. 5,5′-Dithiobis-(2-nitrobenzoic acid) (DTNB) will react with CEA only after reduction of the disulphide bonds with dithioerythritol. Reduction-alkylation of CEA using dithioerythritol and bromo-[1-$^{14}$C] acetic acid confirmed the presence of 6 disulphide bonds, as did oxidation of the glycoprotein with performic acid. The products from the DTNB and reduction-alkylation treatments of CEA had less capacity to inhibit the binding of [$^{125}$I]-CEA to anti-CEA in a radioimmunoassay than the original CEA but could, in sufficient quantities, totally inhibit the binding. Removal, using mercaptoethanol, of the thiol blocking groups from the DTNB-treated CEA resulted in a 55% recovery of antigenic activity. The product from the performic acid oxidation could only inhibit approximately 50% of the binding. Treatment of CEA with 0·533M sodium periodate ($NaIO_4$) greatly reduced its antigenic activity, presumably a result of the oxidative cleavage of the disulphide bonds. No loss in activity, however, was observed when 5·33mM $NaIO_4$ was used, and one Smith degradation (*i.e.* treatment in sequence with periodate, borohydride and mild acid) of CEA removed approximately 50% of the carbohydrate, including all of the fucose, sialic acid and 2-acetamido-2-deoxygalactose but did not change the antigenic activity.**

At the present time, little is known about the precise nature of the immunological determinant(s) of carcinoembryonic antigen (CEA). Evidence for the involvement of 2-acetamido-2-deoxyglucose in the immunodominant grouping has been presented by Banjo *et al.* (1972) and is well supported by further work from the same laboratories (Banjo *et al.*, 1974). In addition, Watanabe and Hakomori (1973) have shown that purified glycosphingolipids from tumour tissue or erythrocytes give a strong precipitin reaction with wheat germ lectin and with anti-CEA. It is known that sialic acid does not form part of the immunological determinant (Banjo *et al.*, 1972; Coligan *et al.*, 1973), nor, according to recent work on the treatment of CEA with periodate, does fucose (see Terry *et al.*, 1974).

Recently, Neveu *et al.* (1975) proposed that CEA and the "nonspecific cross-reacting antigen", NCA, have common antigenic determinants on both the carbohydrate and polypeptide parts of the molecule, while Hammarström *et al.* (1975) conclude, tentatively, that the carbohydrate moiety of CEA does not contain the tumour associated determinant(s). The latter statement concurs with our report (Westwood, Thomas and Foster, 1974*b*) and that of Coligan and Todd (1975) that a large quantity of carbohydrate in CEA can be destroyed using periodate, without causing any loss in antigenic activity.

Whichever parts of the molecule constitute the immunological determinants, carbohydrate or protein or both, it is obvious that disorientation of the determinant groups by cleavage of the protein chain using either proteolytic enzymes

(Banjo *et al.*, 1974) or dilute acid or alkali (Westwood *et al.*, 1974*a*) produces a marked reduction in the ability of the molecule to bind to anti-CEA antiserum.

The work described in this paper was concerned with the determination of the number of disulphide bonds in CEA and an examination of the rôle played by these disulphide bonds and by the carbohydrate component, in the binding of CEA to its antiserum. Evidence is presented for the presence in CEA of 6 intramolecular disulphide bonds, which appear to play an important rôle in holding the CEA molecule in a fixed conformation. Also, we have shown that a considerable part of the carbohydrate can be removed from the glycoprotein using sodium periodate ($NaIO_4$) but the retention of full antigenic activity is dependent on the molarity of the $NaIO_4$ used. The difference appears to be due to a cleavage or non-cleavage of the disulphide bonds. Some of this work has been published in a preliminary form (Thomas, Westwood and Foster, 1974; Westwood *et al.*, 1974*b*).

### MATERIALS AND METHODS

*CEA*.—All preparations of CEA used in this work were isolated from the metastatic liver tumours of patients with colorectal carcinoma using essentially the method described by Krupey, Gold and Freedman (1968). Thus, after extraction of the tumour homogenate with 1·0 mol/l perchloric acid, purification was achieved by chromatography on Sepharose 4B and Sephadex G-200 columns, followed by an acetone precipitation of the CEA from 5% aqueous acetic acid. No electrophoresis step was used. Each CEA sample gave a single band in polyacrylamide gel (10 and 20% in acetic acid, pH 2·4) electrophoresis, a high titre in the radioimmunoassay and values in amino acid and monosaccharide analysis close to those already reported (Turberville *et al.*, 1973; Westwood *et al.*, 1974*a*) for CEA preparations.

*Chemicals*.—Dithioerythritol was purchased from the Sigma Chemical Company, Kingston-upon-Thames, Surrey, England. 5,5′-Dithiobis-(2-nitrobenzoic acid) (DTNB) was obtained from the Aldrich Chemical Company Inc., Milwaukee, Wisconsin, U.S.A; $NaIO_4$ was bought from May and Baker Ltd, Dagenham, England, and sodium borohydride from the British Drug Houses Ltd, Poole, England. Bromo[1-$^{14}$C]acetic acid (40 mCi/mmol) and sodium borotritiide (870 mCi/mmol) were bought from the Radiochemical Centre, Amersham, England. The bromoacetic acid was diluted to a specific activity of 150 μCi/mmol using unlabelled bromoacetic acid and the sodium borotritiide diluted to 40 mCi/mmol using sodium borohydride. All other chemicals were of analytical grade.

*Monosaccharide and amino acid analysis*.—Monosaccharide analysis was carried out according to the method described by Clamp, Bhatti and Chambers (1971), using a Perkin–Elmer F-30 gas chromatograph containing 2, matched 6 ft × 0·25 in glass columns packed with 2·5% silicone gum rubber (E 301) on AW-DMCS Chromosorb G (80–100 mesh). After injection of the samples the temperature of the columns was raised from 120 °C at the rate of 1 °C/min to 210 °C. Mannitol and perseitol were used as internal standards. Sialic acid was determined using Warren's (1959) thiobarbituric acid method. Amino acid analyses were carried out using a Jeol automatic amino acid analyser (JLC 6AH) after hydrolysis using 6N HCl of samples under an atmosphere of nitrogen, at 110 °C for 24 h. Tryptophan was determined using the method of Goodwin and Morton (1946) and the following formula, derived by Beaven and Holiday (1952):

$$M_{Try} = (0{\cdot}263 \times K_{280} - 0{\cdot}170 \times K_{294}) \times 10^{-3},$$

where $K_{294}$ and $K_{280}$ are the extinction coefficients of the protein in 0·1N NaOH at 294 and 280 nm and $M_{Try}$ is the amount of tryptophan (gmol) in 1 g of protein.

*Radioimmunoassay*.—The radioimmunoassay used throughout this work was the double antibody technique described by Laurence *et al.* (1972).

*Liquid scintillation counting*.—This was performed using Packard Tri-Carb liquid scintillation spectrometers (Models 3320 and 3375) and a phosphor mixture containing toluene (1925 ml), dioxan (1925 ml), methanol (1150 ml), naphthalene (400 g) and butyl PBD (35 g).

*Reaction of CEA with bromo*[1-$^{14}$*C*]*acetic acid*.—The procedure used was essentially that described by Königsberg (1972). CEA (2 mg) was dissolved in 1 ml of a 0·15M tris buffer (pH 8·0) containing 0·002M ethylene diamine tetraacetic acid (EDTA) and 6M guanidine hydrochloride. This solution was incubated at 50 °C for 0·5 h and dithioerythritol (60 μmol) added. The solution was purged for 10 min with oxygen-free nitrogen and incubated for a further 4 h at 50 °C. Bromo[1-$^{14}$C]acetic acid (200 μmol, 250 μCi/mmol) was added and the solution again purged with nitrogen and finally incubated at 25 °C for 1·5 h. The solution was then extensively dialysed with water (3 changes of 3 l for 24 h) and freeze-dried. The specific activity of a standard solution of the freeze-dried material was calculated after liquid scintillation counting. Samples were also submitted for radioimmunoassay, amino acid analysis and monosaccharide analysis. Controls in which the dithioerythritol or both dithioerythritol and 6M guanidine hydrochloride were absent were carried out at the same time.

*Oxidation of CEA using performic acid.*—The procedure described is essentially the method of Hirs (1966). CEA (2 mg) was dissolved in formic acid (0·1 ml, 98–100%) and kept at 0 °C. Hydrogen peroxide (30%, 10 μl) was added and the mixture kept at 0°C for 2·5 h. At the end of this period, ice water (2 ml) was added and the mixture was freeze-dried. The dried material was dissolved in water (2 ml) and freeze-dried again. Samples were subjected to amino acid and monosaccharide analysis and also submitted for radioimmunoassay.

*Reaction of CEA with 5,5′-dithiobis (2-nitrobenzoic acid).*—CEA (4 mg) was dissolved in 0·15M Tris-HCl buffer (2 ml, pH 8·0) containing 0·002M EDTA and 6M guanidine hydrochloride. This solution was incubated for 4 h at 50 °C and flushed with nitrogen. A solution of 5,5′-dithiobis(2-nitrobenzoic acid DTNB, 200 μmol in 1 ml of the above buffer) was added and the mixture incubated for a further 0·5 h. The optical density of the solution at 412 nm was measured using a Unicam SP500 spectrophotometer. After dialysis with water, the solution was freeze-dried and the product submitted for radioimmunoassay.

CEA (4 mg) was dissolved in 2 ml of the above buffer and incubated at 50 °C for 0·5 h. The mixture was flushed with nitrogen and dithioerythritol (60 μmol) added. The solution was incubated at 50 °C for a further 4 h when DTNB (500 μmol) in 2 ml of the buffer was added. The solution was then dialysed with two 2 l changes of a 3 mM DTNB solution. The material was further dialysed with four 2 l changes of water, and then freeze-dried. The solid was dissolved in water (3 ml) dialysed with a further 2 l of water freeze-dried again and submitted for radioimmunoassay.

A weighed sample of the solid material was dissolved in 0·15M Tris-HCl buffer (pH 8·0, 1 ml) and 2-mercaptoethanol (10 μl) added. The change in $E_{412}$ produced was measured spectrophotometrically. The solution was then dialysed with a 5 mM solution of 2-mercaptoethanol (2 l) and chromatographed on a column of Sephadex G-200 (1·6× 100 cm). Tubes containing CEA-active material were pooled, the solution freeze-dried and the residue again analysed by radioimmunoassay.

*Molecular weight determinations by gel filtration.*—Two columns, a Sephadex G-200 and a Biogel A-1·5 m (both 1·6 × 100 cm), were prepared and calibrated using a range of standards (Andrews, 1965). The standard proteins and modified CEA samples were eluted from the G-200 columns using a 0·05M phosphate buffer (pH 7·2; containing 0·1% sodium azide) and from the Biogel A-1·5 m column using 0·1M Tris-HCl buffer (pH 7·5, containing 0·2% sodium azide).

*Gel electrophoresis in sodium dodecylsulphate.*—Gel electrophoresis of CEA samples in sodium dodecylsulphate was carried out using the method of Weber and Osborn 1969).

*Sedimentation velocity measurements.*—Sedimentation experiments were performed in a double sector cell (0·36 ml channel at 50740 rev/min in a Beckman Model E Ultracentrifuge. Samples of the glycoprotein to be analysed were dissolved in 0·1M phosphate buffer (pH 7·0). Sodium dodecylsulphate (0·1% or 0·5%) was incorporated for some experiments.

*Quantitative N-terminal analysis.*—The procedure was that of Stark and Smyth (1963). CEA, after performic acid oxidation, was carbamylated overnight with KCNO and 10·4 mg of the carbamylated glycoprotein used for the determination of the *N*-terminal amino acid.

*Treatment of CEA with solutions of $NaIO_4$ of different concentrations.*—Equal weights (800 μg) of CEA were treated with aqueous solutions (110 μl) of $NaIO_4$ of the following molarities: 4·85 × $10^{-1}$M, 4·41 × $10^{-2}$M, 4·01 × $10^{-3}$M and 3·65 × $10^{-4}$M. The solutions were left isolated from light for 20 h at room temperature. After destruction of excess of $NaIO_4$ using ethylene glycol, samples were submitted for radioimmunoassay.

*Treatment of CEA with 0·533*M *$NaIO_4$*.—Solutions of CEA (10 mg/ml) in aqueous or buffered (0·2M acetate buffer, pH 3·8) 0·533M $NaIO_4$ solution were stored in the dark at room temperature for various periods of time. The excess of $NaIO_4$ was destroyed using ethylene glycol and samples were submitted for radioimmunoassay. Amino acid analyses were carried out after dialysis of the solution for 24 h with distilled water and freeze-drying. For one of the analyses extreme care was taken to exclude molecular oxygen from the hydrolytic stage by rigorous purging of the solution with a stream of nitrogen gas in an attempt to detect any cystine in the product.

In one experiment, instead of freeze-drying (which gives an insoluble product) the solution after dialysis, a small volume (350 μl) was added to 50 mM phosphate buffer (pH 7·2) and eluted from a column (100 cm × 1·6 cm) of Sephadex G-200 in the same buffer. The remaining solution was added to an equal volume of 0·2M carbonate-bicarbonate buffer (pH 9·2) and a $3 \times 10^4$ molar excess of sodium borohydride (to CEA) was added. The solution was left at room temperature for 6 h and then dialysed for 24 h with distilled water and freeze-dried. Monosaccharide analysis was carried out on the product and the extent to which it inhibited, at different concentrations, the binding of [$^{125}$I]CEA to anti-CEA in the radioimmunoassay, was measured.

*Treatment with 5·33 m*M *$NaIO_4$ and Smith degradation of CEA*.—Solutions of CEA (1 mg/ml in aqueous or buffered (0·2M acetate buffer, pH 3·8) 5·33 mM $NaIO_4$ solution were stored in the dark at room temperature for times up to 44 h. Radioimmunoassays and amino acid analyses were carried out exactly as described for the above treatment with 0·533M $NaIO_4$. Also, chromatography on Sephadex G-200 and reduction of the product using sodium borohydride and examination of the reduced product were carried out as described above.

For the complete Smith degradation (Goldstein *et al.*, 1965) the solution from the $NaIO_4$ oxidation was dialysed with distilled water for 24 h and its pH then adjusted to 9·2 with 0·1M NaOH solution. After mixing this solution with an equal volume of 0·2M carbonate-bicarbonate buffer (pH 9·2), sodium borotritiide (40 mg, specific activity 40 mCi/mmol) was added and the solution left at room temperature for 6 h and then dialysed for 3 days with distilled water. The product was freeze-dried, its inhibitory activity in the radioimmunoassay and its specific activity (in μCi/mg) were determined.

A solution of the above product (5·7 mg) in 0·1N $H_2SO_4$ (2 ml) was kept at 37 °C for 22 h. After dialysis with frequent changes of distilled water for 24 h and freeze-drying, the inhibitory activity in the radioimmunoassay and the specific activity (in μCi/mg) of the product were determined. Monosaccharide and amino acid analyses were carried out.

*Treatment of CEA sequentially with sodium borotritiide and 0·1*N *$H_2SO_4$*.—A solution of CEA (2·54 mg) in carbonate-bicarbonate buffer (pH 9·2) containing dissolved sodium borotritiide (6·5 mg, specific activity 40 mCi/mmol) was left for 6 h at room temperature. The solution was then dialysed with distilled water for 36 h with frequent changes of water and then freeze-dried. The specific activity of this material was determined.

The remaining product (1·7 mg) was dissolved in 0·1N $H_2SO_4$ and kept for 22 h at 37 °C. After dialysis with water for 24 h the solution was freeze-dried and the product's specific activity and inhibitory activity in the radioimmunoassay were determined.

### RESULTS

### *Treatment of CEA with bromo-*[1-$^{14}$*C*] *acetic acid, performic acid and DTNB*

When bromo-[1-$^{14}$C]acetic acid was reacted with CEA under reducing and denaturing conditions, 16·7 mol of $^{14}$C were incorporated per mol of CEA. Reaction of native CEA with bromo-[1-$^{14}$C]acetic acid in the absence of dithioerythritol and in the absence or presence of 6M guanidine hydrochloride resulted in the incorporation of 4·8 mol and 5·2 mol respectively of $^{14}$C per mol of CEA. Subsequent amino acid analysis of the products showed the presence of 10·8 mol of carboxymethyl cysteine per mol of CEA from the sample reacted under denaturing conditions while in the alkylated native CEA there was no evidence for the presence of carboxymethyl cysteine. The site of labelling in the native CEA could not be identified. No other changes in the amino acid composition of CEA could be observed and the carbohydrate analysis was unchanged. Gel filtration on Sephadex G-200 showed that the molecular size of the reduced, alkylated CEA was of the same order as that of native CEA.

When CEA was oxidized with performic acid, amino acid analysis of the product showed the presence of 11·5 mol of cysteic acid per mol of CEA. There was no modification of other amino acids except tryptophan (88% loss) and the carbohydrate analysis was identical to that of native CEA. The oxidized material also behaved similarly to native CEA on gel filtration.

After reaction with DTNB under reducing conditions followed by disulphide exchange with 2-mercaptoethanol, colorimetric estimations of liberated 5-thio-2-nitrobenzoate showed the presence of 12·2 mol of sulphide (as cysteine) per mol of CEA. Reaction of CEA with DTNB without prior reduction confirmed the absence of any free cysteine. Gel filtration of DTNB-modified CEA again showed no gross change in molecular size. The results from these experiments are summarized in Table I. The results from the amino acid and monosaccharide analyses are shown in Table II.

Sodium dodecylsulphate electrophoresis in polyacrylamide gels of native and performic acid oxidized CEA failed to demonstrate any changes in the electrophoretic mobility of the performic acid oxidized material.

Determination of the sedimentation velocities ($S_{20,w}$) of native and performic acid oxidized CEAs, however, showed striking differences. Native CEA had a $S_{20,w}$ value of 6.21 while the modified material had a $S_{20,w}$ value of only 3·80. In the presence of 0·1% SDS, the $S_{20,w}$ value for the oxidized CEA remained the same while a second component with an $S_{20,w}$ value of 3·5 was observed with the native CEA sample. Increasing the SDS concentration to 0·5% again resulted in two components being observed for the native CEA with $S_{20,w}$ values of 4·9 and 3·3 respectively. These results are not indicative of a gross molecular weight change in the oxidized CEA but rather a gross change in the overall conformation of the molecule, extended molecules tending to sediment more slowly than more compact molecules (Bais *et al.*, 1974).

TABLE I.—*The Disulphide Bridges of Carcinoembryonic Antigen*

| Modification | No. mol of -SH reacting/ mol CEA (2·2 × $10^5$) | Mol. wt. of modified CEA by gel filtration | RIA‖ value (%) |
|---|---|---|---|
| None (native CEA) | — | 2·2 × $10^5$ | 100 |
| Bromo-[1-$^{14}$C)acetic acid alone | 0 | — | 100 |
| Bromo-[1-$^{14}$C]acetic acid + 6M guanidine HCl | 0 | — | 100 |
| Bromo-[1-$^{14}$C]acetic acid + 6M guanidine HCl + DTE§ | 11·5, 11·9* 10·8† | 2·8 × $10^5$ | 8 |
| Performic acid | 11·5‡ | 2·8 × $10^5$ | 10 |
| DTNB in non-reducing conditions | 0 | 2·2 × $10^5$ | 100 |
| DTNB + DTE | 12·2 | 2·8 × $10^5$ | 15 |
| Disulphide exchange of DTNB-CEA with mercaptoethanol | — | 2·3 × $10^5$ | 55 |

* By incorporation of label—this value represents the difference between the number of mol of label incorporated in the presence of DTE and 6M guanidine HCl (16·7) and the number of mol of label incorporated in the absence of DTE and in the presence (5·2) or absence (4·8) respectively of 6M guanidine HCl.

† By amino acid analysis for carboxymethylcysteine.

‡ By amino acid analysis for cysteic acid.

§ Dithioerythritol.

‖ Radioimmunoassay.

TABLE II.—*Amino acid and Monosaccharide Analyses* of Performic Acid Oxidized and Reduced Alkylated CEA*

| | CEA | Performic acid oxidized CEA | Reduced alkylated CEA |
|---|---|---|---|
| CM Cyst | — | — | 1·2 |
| Cys. ac. | 0·6 | 1·5 | — |
| Asp | 14·6 | 15·0 | 14·9 |
| Thr | 9·4 | 9·6 | 9·6 |
| Ser | 10·4 | 10·8 | 10·6 |
| Glu | 10·4 | 10·3 | 10·5 |
| Pro | 8·1 | 8·2 | 8·2 |
| Gly | 5·4 | 5·6 | 5·7 |
| Ala | 6·1 | 6·1 | 6·1 |
| Val | 7·2 | 7·1 | 7·1 |
| Cys | none | none | none |
| Met | trace | none | none |
| Ile | 5·1 | 5·0 | 5·2 |
| Leu | 8·7 | 8·6 | 8·3 |
| Tyr | 3·7 | 2·9 | 3·3 |
| Phe | 2·3 | 2·5 | 2·4 |
| Lys | 2·7 | 2·6 | 2·5 |
| His | 1·9 | 1·9 | 1·5 |
| Arg | 3·4 | 2·3 | 2·9 |
| Fucose | 20 | 23 | 23 |
| Mannose | 10 | 9 | 9 |
| Galactose | 22 | 22 | 23 |
| GalNAc | 1 | 1 | 1 |
| GlcNAc | 43 | 44 | 44 |
| Sialic acid | 4 | n.d.† | n.d. |

* Values are expressed as mol percentages, separately for amino acids and monosaccharides.
† n.d. not determined.

Quantitative *N*-terminal analysis of CEA confirmed the likelihood that the molecule consists of only a single polypeptide chain by showing lysine as the major *N*-terminal (~90%) with 0·8 mol of the amino acid per mol of CEA.

The variously modified CEAs were then analysed with respect to their ability to bind to antibody directed against native CEA. Inhibition curves for native CEA, performic acid oxidized CEA and reduced and alkylated CEA are shown in Fig. 1.

The modified antigens have a reduced activity in the radioimmunoassay and complete inhibition of labelled antigen–antibody binding is never achieved in the case of the performic acid oxidized material.

CEA, reacted with DTNB, also had a much reduced activity in the radioimmunoassay, about 10% of that of native CEA. However, after disulphide exchange with 2-mercaptoethanol and gel filtration to remove released 5-thio-2-nitrobenzoate the isolated antigen had recovered approximately 55% of the activity of the original CEA.

*Treatment of CEA with solutions of $NaIO_4$ of different concentrations*

Table III shows the titres in the radioimmunoassay of the solutions of CEA left for 20 h at room temperature in different concentrations of $NaIO_4$. In only one case, that involving 0·485M $NaIO_4$, was the titre below that of the

TABLE III.—*RIA* values in different concentrations of $NaIO_4$*

| Concentration of $NaIO_4$ (M) | RIA value (ng/ml) |
|---|---|
| $3·65 \times 10^{-4}$ | 85·0 |
| $4·01 \times 10^{-3}$ | 87·0 |
| $4·41 \times 10^{-2}$ | 73·0 |
| $4·85 \times 10^{-1}$ | 17·5 |
| 0 | 64·25 |

* Radioimmunoassay.

FIG. 1.—Inhibition of [$^{125}$I]-CEA-anti-CEA binding. Normal CEA standard curve (▲); performic acid oxidized CEA (●); reduced-alkylated (bromo-[1-$^{14}$C]acetic acid) CEA (■); CEA treated with 5·33 mM $NaIO_4$ and sodium borohydride (○); Smith degraded CEA (△); CEA treated with 0·533M $NaIO_4$ and sodium borohydride (□).

control. The other 3 $NaIO_4$ solutions elevated the titre in the radioimmunoassay.

*Treatment of CEA with 0·533M $NaIO_4$*

When a solution of CEA (10 mg/ml) in 0·533M $NaIO_4$ (12·5 mol/mol of monosaccharide) was left at 20 °C in the absence of light, the titre in the RIA fell, after 22 h, 43 h and 66 h to 11·4%, 3·5% and 1·0% respectively of the original value. The dialysed, freeze-dried material proved to be insoluble in water, *N* aqueous sodium hydroxide and *N* aqueous acetic acid. Its total amino acid analysis (see Table IV) showed a loss of nearly all the tyrosine, approximately one-third of the arginine and possibly small amounts of lysine and histidine but, otherwise, little difference from that of the original CEA. From the analysis in which precautions were taken to exclude molecular oxygen during the hydrolysis stage, no cystine could be detected in the material whereas a quantity of cysteic acid, equivalent to approximately 7 mol per mol of CEA, were detected.

Chromatography of the product from the oxidation on Sephadex G-200 produced a peak of activity in the radioimmunoassay at an elution volume corresponding to a mol. wt of ~200,000.

A curve representing the inhibition by the reduced, modified CEA of the binding of [$^{125}$I]-CEA to anti-CEA in the radioimmunoassay is given in Fig. 1. A concentration of 18 μg/ml was required for an inhibition of 50%.

*Treatment of CEA with 5·33 mM $NaIO_4$*

The radioimmunoassay titre of equal aliquots of a solution of CEA (1 mg/ml) in 5·33 mM $NaIO_4$ (1·3 mol/mol of monosaccharide) had increased after 24 h to a value 8·3% greater than the value at the beginning of the experiment but after 44 h had fallen back to the original. Dialysis of the solution with water for 24 h and freeze-drying produced an insoluble product whose total amino acid analysis is shown in Table IV. As in the oxidation using 0·533M $NaIO_4$, destruction of tyrosine and arginine and possibly small amounts of lysine and histidine was

TABLE IV.—*Amino Acid Analyses of CEA and Modified CEA Samples* (mol/100 mol Amino Acids)

| | a* | b | c | d | e |
|---|---|---|---|---|---|
| Cys. ac | 0·6 | 1·5 (0·25)† | 1·5 (0·9)† | none | none |
| Asp | 14·6 | 15·3 | 16·4 | 15·3 | 15·3 |
| Thr | 9·4 | 9·5 | 10·3 | 9·9 | 9·6 |
| Ser | 10·4 | 10·7 | 10·9 | 10·5 | 11·0 |
| Glu | 10·4 | 11·0 | 11·0 | 10·6 | 11·1 |
| Pro | 8·1 | 8·3 | 7·6 | 9·4 | 8·7 |
| Gly | 5·4 | 6·1 | 6·0 | 5·5 | 5·6 |
| Ala | 6·1 | 6·3 | 6·4 | 6·0 | 6·1 |
| Val | 7·2 | 7·0 | 7·0 | 7·1 | 7·1 |
| Cys | none | none (0·85)† | none (0)† | none | none |
| Met | trace | none | none | none | none |
| Ile | 5·1 | 4·9 | 4·8 | 4·5 | 4·6 |
| Leu | 8·7 | 8·4 | 8·5 | 8·4 | 8·5 |
| Tyr | 3·7 | 1·3 | trace | 3·2 | 2·7 |
| Phe | 2·3 | 2·5 | 2·4 | 2·4 | 2·4 |
| Lys | 2·7 | 2·3 | 2·4 | 1·9 | 1·9 |
| His | 1·9 | 1·6 | 1·6 | 1·5 | 1·6 |
| Arg | 3·4 | 2·6 | 2·3 | 2·8 | 3·3 |

a, CEA; b, CEA treated with 5·33 mM sodium periodate; c, CEA treated with 0·533M sodium periodate; d, CEA treated with 5·33 mM sodium periodate-sodium borohydride; e, product from one Smith degradation of CEA.

* Analysis results from Turberville *et al.* (1973).

† Values obtained after rigorous de-oxygenation of solution for hydrolysis.

observed but otherwise the $NaIO_4$ appeared to have had little effect on the protein part of the molecule. In the amino acid analysis in which precautions were taken to exclude molecular oxygen in the hydrolysis stage, both cystine and cysteic acid were detected in amounts equivalent to 7 mol of cysteine and 2 mol of cysteic acid per mol of CEA. Chromatography on Sephadex G-200 of a solution of the oxidized material, after dialysis but before the freeze-drying stage, eluted at a volume corresponding to a mol. wt of approximately 200,000.

Reduction of the products from the 44 h $NaIO_4$ treatment of different samples of CEA with sodium borohydride gave soluble materials. On monosaccharide analysis they were found to have lost (average of 4 samples), compared with the original amounts of monosaccharides, all of the fucose, 2-acetamido-2-deoxygalactose and sialic acid, 15% of the mannose, 50% of the galactose and 3% of the 2-acetamido-2-deoxyglucose. Analysis (see Table IV) of the material showed that the amino acid composition was still almost the same as that of CEA.

A curve depicting the inhibition of [$^{125}$I]-CEA/anti-CEA binding by the modified CEA is given in Fig. 1. A concentration of 74 ng/ml was required for 50% inhibition.

*Smith degradation of CEA*

The product obtained from the reduction of the oxidized CEA using sodium borotritiide had a specific activity of 33·82 μCi/mg and its inhibition curve, as measured in the radioimmunoassay, was identical to that of the material obtained at the corresponding stage of the degradation when sodium borohydride was used instead of sodium borotritiide (see above).

Figure 2 shows the accumulation of radioactivity outside the dialysis bag during an experiment designed to measure the rate at which the labelled, degraded fragments of sugar are eliminated by the dilute acid treatment. The modified CEA from this treatment was found to be still radioactive, with a specific activity of 19·65 μCi/mg. Its inhibition curve in the radioimmunoassay is shown in Fig. 1. Thus a concentration of 64 ng/ml was

FIG. 2.—Elimination of tritium label during acid treatment stage of Smith degradation of CEA. A solution of the sodium borotritiide reduced material in 0·1N $H_2SO_4$ was contained inside a dialysis bag. This was dialysed with 0·1N $H_2SO_4$ at 37 °C and aliquots of the solution outside the bag were removed at noted times and the amounts of radioactivity determined.

required for 50% inhibition. On chromatography of the product using water on a Biogel P-10 column, all the radioactivity was excluded from the column and was shown by radioimmunoassay to be associated with the material which would inhibit the [$^{125}$I]-CEA/anti-CEA binding. A comparison between the values from the monosaccharide analysis of this product

TABLE V.—*Monosaccharide Analyses* (*in μmol/100 mg of glycoprotein*) *of Smith Degraded CEA*

| | CEA | Smith degraded CEA |
|---|---|---|
| Fucose | 51·8 | none |
| Mannose | 24·6 | 21·1 |
| Galactose | 63·9 | 36·9 |
| GalNAc* | 1·0 | none |
| GlcNAc† | 91·5 | 111·4 |
| Sialic acid | 5·6 | none |

* 2-acetamido-2-deoxygalactose.
† 2-acetamido-2-deoxyglucose.

and the CEA from which it was derived is shown in Table V. Thus fucose, 2-acetamido-2-deoxygalactose and sialic acid were absent in the modified material. As the figures in Table V are expressed in μmol/100 mg of glycoprotein, calculations of percentage amounts of monosaccharides lost must take into account that approximately 50% of the carbohydrate is destroyed on periodate oxidation. On this basis, this particular sample of CEA lost 26% mannose, 57% galactose and 9% 2-acetamido-2-deoxyglucose. Amino acid analysis (Table IV) showed a reduction in tyrosine content (~30%) and small losses of lysine and histidine compared with the original CEA. No cysteic acid could be detected in the modified material. A determination of tryptophan in this material showed a decrease from 10·5 mol/mol of glycoprotein in the original CEA to 6·15 mol/mol of glycoprotein in the Smith degraded material. Again, allowing for the loss of carbohydrate from the material, this represents a loss of tryptophan from the protein part of the glycoprotein of 61%.

*Treatment of CEA sequentially with sodium borotritiide and 0·1N $H_2SO_4$*

The direct treatment of CEA with sodium borotritiide produced, after dialysis with distilled water and freeze-drying, a product of specific activity 9·68 μCi/mg. Subsequent treatment of this material with 0·1N $H_2SO_4$ for 22 h at 37 °C (as in the Smith degradation) gave a product, still labelled (specific activity 8·57 μCi/mg) and which had retained full antigenic activity, requiring a concentration of 49 ng/ml for 50% inhibition in the radioimmunoassay.

### DISCUSSION

Any discussion of the immunogenic groups of material as complex as CEA is necessarily limited in relation to the antiserum used to measure such activity, until it has been shown that all antisera raised against the material are directed against the same part(s) of the molecule. In our attempts to relate the different binding capacities to the changes effected in the CEA molecule by chemical and enzymatic means, we have so far used only a goat anti-CEA antiserum. Previous work (Westwood *et al.*, 1974*a*) showed that when using this particular system, a large percentage of the antigenic activity of CEA is lost by cleavage of the protein chain using either chemical (0·05N NaOH or 0·01N $H_2SO_4$) or enzymatic (pepsin) methods. It was recognized that such chemical agents as alkali and acid have effects on CEA other than cleavage of the protein chain and we have now used reagents more discriminate in their action on the glycoprotein.

The presence of cysteic acid in the amino acid analysis (Turberville *et al.*, 1973) of CEA prompted an examination of the nature of the involvement of cysteine in the molecule. The use of bromo-[I-$^{14}$C] acetic acid with 6M guanidine hydrochloride in the absence and presence of dithioerythritol indicated that CEA contained no free cysteine but, rather, cystine in the form of 6 disulphide bonds. Further evidence for this structural feature was obtained by oxidation of CEA using performic acid and determining the amount of cysteic acid (11·5 mol/mol CEA) produced. Apart from the oxidation of the cystine residues to cysteic acid and an 88% reduction in tryptophan, this reagent did not alter the composition of CEA in any other way. However, a loss of 61% of the tryptophan after periodate oxidation did not result in any loss in antigenic activity.

Of particular interest was the reaction of CEA with DTNB, which enabled not only a confirmation of the presence of 6 disulphide linkages in the molecule, but also a direct means of illustrating the involvement of these linkages in the binding to the antiserum. Thus, the CEA carrying the 5-thio-2-nitrobenzoate groups had an activity in the radioimmunoassay of only 10% of the activity of the native CEA whereas on removal of these groups the CEA regained 50–55% of its original activity. That the activity did not return fully to its original value is presumably due to the failure of the disulphide bonds to rejoin completely in the original conformation.

A common feature of the products from the experiments involving cleavage of the disulphide bonds, as described above, was that they had lost most of the antigenic activity of the original CEA. An additional point of interest was that if sufficient quantities were used, the product from the reduction–alkylation experiment produced virtually maximum inhibition in the radioimmunoassay whereas the product from the performic acid oxidation, which is unchanged in composition (except for cystine and tryptophan) never produced more than 45% inhibition. This result is surprising as it suggests that destruction of important determinant groups occurred. An explanation suggesting that the antiserum contains a population of antibodies which only bind CEA in its native conformation ought equally to apply to the reduced-alkylated product.

The change in shape of the CEA molecule when the disulphide bonds were broken was manifested in the measurements of sedimentation velocities. Quantitative *N*-terminal analysis showed that CEA almost certainly contains a single polypeptide chain and gel filtration of the products in which the disulphide bonds had been broken showed that no gross change in molecular size had occurred. However, in sedimentation velocity measurements CEA behaved as a much more compact molecule than did the material from the performic acid oxidation.

Schmid *et al.* (1974) recently determined the positions of the 2 disulphide bonds in the polypeptide chain of $\alpha_1$-acid glycoprotein. We believe that a situation similar to the one represented by their schematic diagram exists in CEA with 6 disulphide bonds cross-linking a long single polypeptide chain.

The reduction of the antigenic activity of CEA by cleavage of the disulphide bonds does not, of course, give any information on the nature of the determinant groups as it could merely result from a disorientation of groups so as to seriously diminish the antigen–antibody binding.

A possible way of investigating the role of the carbohydrate part of the molecule in the antigenic activity is to use a reagent, such as $NaIO_4$, which under suitable conditions will preferentially destroy carbohydrate, in this case all monosaccharides containing vicinal diol groups, so including all terminal sugars, but leave the protein less damaged. The oxidizing conditions (pH 3·8, room temperature, *i.e.* ~20°) were chosen to give a reasonably rapid oxidation of the sugars without overoxidation (Jeanloz and Forchielli, 1951). At 0·533M periodate destroyed a large part of the antigenic activity of CEA whereas at 5·33 mM no change in activity occurred. The latter conditions destroyed (average of 4 samples) all the fucose, 2-acetamido-2-deoxygalactose, and sialic acid, 15% of the mannose, 50% of the galactose and 3% of the 2-acetamido-2-deoxyglucose.

The loss of activity in the stronger $NaIO_4$ solution is probably due to cleavage of the disulphide bonds. Only cysteic acid could be detected in this product but a considerable amount of cystine was found in the 5·33 mM oxidation product. Completion of the Smith degradation yielded a product retaining full antigenic activity but having lost approximately 50% of its carbohydrate. This meant that the terminal monosaccharides of CEA play no part in its antigenic activity. It is much more probable that the determinant groups reside on the polypeptide.

During the Smith degradation the polypeptide lost 61% of its tryptophan and approximately 30% of its tyrosine.

Clamp and Hough (1965) have reported the destruction of some amino acids in 13 mM periodate. Thus the isolation of an unmodified polypeptide chain using this method is not possible. Tyrosine and tryptophan are attacked even at very low concentrations of periodate whereas the cleavage of the disulphide bonds of cystine requires higher concentrations.

It has therefore become clear that the breaking of the disulphide bonds of CEA causes a large loss of antigenic activity. In the case of cleavage with performic acid, there seems to be associated destruction of important antigenic determinant(s). This observation has prompted a potentially useful modification of the CEA assay involving absorption of the normal anti-CEA antiserum with the material from the performic acid oxidation of CEA. The results obtained using this absorbed antiserum will be reported later.

The authors thank Professor A. B. Foster and Dr E. M. Bessell for valuable discussions, Miss S. J. Pelly and Dr C. Turberville for generous supplies of CEA, Miss S. J. Pelly for radioimmunoassays,

Miss R. M. Carter for amino acid analyses, Dr M. A. Bukhari for monosaccharide analyses and Dr K. V. Shooter for the ultracentrifuge runs. The work was supported by the Medical Research Council (Grant No. G973/785/K). The Alexander Keiller Foundation is acknowledged for a fellowship to Dr P. Thomas.

REFERENCES

ANDREWS, P. (1965) The Gel-Filtration Behaviour of Proteins related to their Molecular Weights over a Wide Range. *Biochem. J.*, **96**, 595.

BAIS, R., GREENWELL, P., WALLACE, J. C. & KEECH, D. B. (1974) Influence of Sodium Dodecyl Sulphate on the Sedimentation Velocity of Proteins. *FEBS Letters*, **41**, 53.

BANJO, C., GOLD, P., GEHRKE, C, W., FREEDMAN, S. O. & KRUPEY, J. (1974) Preparation and Isolation of Immunologically Active Glycopeptides from Carcinoembryonic Antigen. *Int. J. Cancer*, **13**, 151.

BANJO, C., GOLD, P., FREEDMAN, S. O. & KRUPEY, J. (1972) Immunologically Active Heterosaccharides of Carcinoembryonic Antigen of Human Digestive System. *Nature, New Biol*,, **238**, 183.

BEAVEN, G. H. & HOLIDAY, E. R. (1952) Ultraviolet Absorption Spectra of Proteins and Amino Acids. *Adv. protein Chem.*, **7**, 319.

CLAMP, J. R., BHATTI, T. & CHAMBERS, R. E. (1971) The Determination of Carbohydrate in Biological Materials by Gas Liquid Chromatography. *Meth. biochem. Anal.*, **19**, 229.

CLAMP, J. R. & HOUGH, L. (1965) The Periodate Oxidation of Amino Acids with Reference to Studies on Glycoproteins. *Biochem. J.*, **94**, 17.

COLIGAN, J. E., HENKART, P. A., TODD, C. W. & TERRY, W. D. (1973) Heterogeneity of the Carcinoembryonic Antigen. *Immunochemistry*, **10**, 591.

COLIGAN J. E. & TODD, C. W. (1975) Structural Studies on Carcinoembryonic Antigen. Periodate Oxidation. *Biochemistry* **14**, 805.

GOLDSTEIN, I. J., HAY, G. W., LEWIS, B. A. & SMITH, F. (1965) Controlled Degradation of Polysaccharides by Periodate Oxidation, Reduction and Hydrolysis. *Meth. carbohyd. Chem.*, **5**, 361.

GOODWIN, T. W. & MORTON, R. A. (1946) The Spectrophotometric Determination of Tyrosine and Tryptophan in Proteins. *Biochem. J.*, **40**, 628.

HAMMARSTRÖM S., ENGVALL, E., JOHANSSON, B. G., SVENSSON, S., GORAN, S. & GOLDSTEIN, I. J. (1975) Nature of the Tumor-associated Determinant(s) of Carcinoembryonic Antigen. *Proc. natn. Acad. Sci. U.S.A.*, **72**, 1528.

HIRS C. H. W. (1966) Performic acid Oxidation. *Meth. Enzymol.*, **11**, 197.

JEANLOZ, R. W. & FORCHIELLI, E. (1951) Studies on Hyaluronic Acid and Related Substances. II. Periodate Oxidation of Glucosamine and Derivatives. *J. biol. Chem.*, **188**, 361.

KÖNIGSBERG, W. (1972) Reduction of Disulphide Bonds in Proteins with Dithiothreitol. *Meth. Enzymol.*, **25**, 185.

KRUPEY, J., GOLD P. & FREEDMAN, S. O. (1968) Physicochemical Studies of the Carcinoembroyonic Antigens of the Human Digestive System *J. exp. Med.* **128**, 387.

LAURENCE D. J. R. STEVENS U. BETTELHEIM R. DARCY, D. A., LEESE, C. L., TURBERVILLE, C., ALEXANDER, P., JOHNS, E. W. & NEVILLE, A. M. (1972) Rôle of Plasma Carcinoembryonic Antigen in Diagnosis of Gastrointestinal, Mammary and Bronchial Carcinoma. *Br. med. J.* iii, 605.

NEVEU, T., STAEBLER, D., CHAVANEL, G. & BURTIN, P. (1975) Study of the Antigenic Cross Reactivity Between Carcinoembryonic Antigen and "Non-specific Cross Reacting Antigens" (NCA and NCA2). *Br. J. Cancer*, **31**, 524.

SCHMID, K., BÜRGI, W., COLLINS, J. H. & NANNO, S. (1974) The Disulphide Bonds of $\alpha_1$ Acid Glycoprotein. *Biochemistry*, **13**, 2694.

STARK, G. R. & SMYTH, D. G. (1963) The Use of Cyanate for the Determination of $NH_2$-terminal Residues in Proteins. *J. biol. Chem.*, **238**, 214.

TERRY, W. D., HENKART, P. A., COLIGAN, J. E. & TODD, C. W. (1974) Carcinoembryonic Antigen: Characterization and Clinical Applications. *Transplantn. Rev.*, **20**, 100.

THOMAS, P., WESTWOOD, J. H. & FOSTER, A. B. (1974) The Rôle of Disulphide Bridges in the Structure and Immunological Activity of the Carcinoembryonic Antigen. *Biochem. Soc. Trans.*, **2**, 1248.

TURBERVILLE, C., DARCY, D. A., LAURENCE, D. J. R., JOHNS, E. W. & NEVILLE, A. M. (1973) Studies on Carcinoembryonic Antigen (CEA) and a Related Glycoprotein CCEA-2. Preparation and Chemical Characterization. *Immunochemistry*, **10**, 841.

WARREN, L. (1959) The Thiobarbituric Acid Assay of Sialic Acids. *J. biol. Chem.*, **234**, 1971.

WATANABE, K. & HAKOMORI, S. (1973) A Glycosphingolipid Sharing Reactivity with Both Wheat Germ Lectin and "Carcinoembryonic Antisera (Gold)": Partial Identity of These Reactive Sites. *FEBS Letters*, **37**, 317.

WEBER, K. & OSBORN, M. (1969) The Reliability of Molecular Weight Determinations by Dodecyl Sulphate-Polyacrylamide Gel Electrophoresis. *J. biol. Chem.*, **244**, 4406.

WESTWOOD, J. H., BESSELL, E. M., BUKHARI, M. A., THOMAS, P. & WALKER, J. M. (1974*a*) Studies on the Structure of the Carcinoembryonic Antigen-1. Some Deductions on the Basis of Chemical Degradations. *Immunochemistry*, **11**, 811.

WESTWOOD, J. H., THOMAS, P. & FOSTER, A. B. (1974*b*) The Treatment of Carcinoembryonic Antigen with Sodium Metaperiodate. *Biochem. Soc. Trans.* **2**, 1250.